# Context-Dependent Regulation of Autophagy by IKK-NF-****κ****B Signaling: Impact on the Aging Process

**DOI:** 10.1155/2012/849541

**Published:** 2012-07-26

**Authors:** Antero Salminen, Juha M. T. Hyttinen, Anu Kauppinen, Kai Kaarniranta

**Affiliations:** ^1^Department of Neurology, Institute of Clinical Medicine, University of Eastern Finland, P.O. Box 1627, 70211 Kuopio, Finland; ^2^Department of Neurology, Kuopio University Hospital, P.O. Box 1777, 70211 Kuopio, Finland; ^3^Department of Ophthalmology, Institute of Clinical Medicine, University of Eastern Finland, P.O. Box 1627, 70211 Kuopio, Finland; ^4^Department of Ophthalmology, Kuopio University Hospital, P.O. Box 1777, 70211 Kuopio, Finland

## Abstract

The NF-**κ**B signaling system and the autophagic degradation pathway are crucial cellular survival mechanisms, both being well conserved during evolution. Emerging studies have indicated that the IKK/NF-**κ**B signaling axis regulates autophagy in a context-dependent manner. IKK complex and NF-**κ**B can enhance the expression of Beclin 1 and other autophagy-related proteins and stimulate autophagy whereas as a feedback response, autophagy can degrade IKK components. Moreover, NF-**κ**B signaling activates the expression of autophagy inhibitors (e.g., A20 and Bcl-2/xL) and represses the activators of autophagy (BNIP3, JNK1, and ROS). Several studies have indicated that NF-**κ**B signaling is enhanced both during aging and cellular senescence, inducing a proinflammatory phenotype. The aging process is also associated with a decline in autophagic degradation. It seems that the activity of Beclin 1 initiation complex could be impaired with aging, since the expression of Beclin 1 decreases as does the activity of type III PI3K. On the other hand, the expression of inhibitory Bcl-2/xL proteins increases with aging. We will review the recent literature on the control mechanisms of autophagy through IKK/NF-**κ**B signaling and emphasize that NF-**κ**B signaling could be a potent repressor of autophagy with ageing.

## 1. Introduction

One part of the aging process involves a decline in cellular housekeeping functions disturbing the maintenance of organism homeostasis [1, 2]. The accumulation of damaged and defective components increases cellular stress, for example, oxidative stress, which activates cellular defence mechanisms including NF-*κ*B signaling pathway and innate immunity system, such as inflammasomes [3–5] (Section 3.1). Aging is associated with a low-grade proinflammatory phenotype which further interferes with housekeeping and cellular homeostasis. Recent studies have indicated that autophagy is a crucial cleansing system preventing inflammation but its capacity clearly declines with aging [6–8]. The NF-*κ*B signaling system and the autophagic degradation pathway have been closely conserved during evolution and emerging studies indicate that these systems have many context-dependent interactions with each other. We will review the recent literature on the control mechanisms of autophagy by NF-*κ*B signaling and particularly we will focus on its context-dependent regulation during the aging process. 

### 1.1. Autophagy

 By definition, autophagy or autophagocytosis is a cellular self-digestion process involving the uptake of cellular components and organelles for degradation by lysosomal system. The hallmark of autophagy is the appearance of autophagosomes which subsequently fuse with primary lysosomes to form autolysosomes where the ingested material is digested by lysosomal enzymes. This process is called macroautophagy, hereafter entitled autophagy. There also exist microautophagy and chaperone-mediated autophagy which deliver cytoplasmic material directly into the lysosomes without the formation of autophagosome. Several reviews have detailed the mechanisms involved in autophagosome formation, for example, over thirty autophagy-related (ATG) proteins are known to participate in this process [9–11]. 

Beclin 1 and its interactome, that is, proteins interacting with Beclin 1, have a crucial role in the vesicle nucleation during autophagosome formation [12, 13]. Beclin 1 and hVPS34, a class III phosphatidylinositol-3 kinase, assemble the core complex which then cooperates with several stable and transient binding partners in order to establish phagophore formation [12]. Autophagosomes most likely originate in the specialized subdomains of endoplasmic reticulum but in addition, Golgi complex and endosomes seem to supply some lipids and proteins to elongating autophagosome membranes [10]. Atg14L protein targets Beclin 1/hVps34 complex to the cup-shaped protrusion of the endoplasmic reticulum, called omegasome [14]. Beclin 1 is a BH3-only protein which can interact with the antiapoptotic Bcl-2 and Bcl-xL proteins which inhibit the autophagosome formation in the initiation phase [15]. However, Bcl-2/xL proteins can be phosphorylated by JNK1, induced by, for example, starvation and ceramides, which block its inhibitory binding to Beclin 1 [13, 16]. Moreover, competitive binding of Bcl-2/xL to other BH3-only proteins, such as BNIP3 and BAD, also prevents Beclin 1 inhibition and induces autophagy [13, 17]. Interactions between Beclin 1 and antiapoptotic Bcl-2/xL proteins are known to regulate the crosstalk between autophagy and apoptosis [18]. Another important regulatory complex in addition to Beclin 1 is the ULK1/2 complex which is controlled by mTOR kinase [10, 19]. mTOR is a potent inhibitor of the ULK1 complex and thus also represses autophagy. There are several extensive reviews on the regulation of autophagy and its physiological and pathological functions [19–22].

### 1.2. IKK-NF-*κ*B Signaling

 NF-*κ*B signaling pathway is involved in the regulation of multiple cellular functions, for example, apoptosis, autophagy, cellular proliferation, differentiation, metabolism, and adaptive and innate immunity responses. The proteins of three families lie in the center of inducible NF-*κ*B signaling module, (i) the IKK kinase complex including IKK*α*, IKK*β*, and IKK*γ* (also called NEMO) are upstream activators of NF-*κ*B signaling, (ii) the NF-*κ*B/Rel DNA-binding complexes involving the NF-*κ*B proteins p50 and p52 and the Rel family members (RelA/p65, RelB, and c-Rel), and (iii) several typical and atypical inhibitory I*κ*B proteins (I*κ*B*α*, I*κ*B*β*, I*κ*B*ε*, I*κ*B*ζ*, I*κ*B*η*, I*κ*BNS, Bcl-3, p100, and p105) [23–25]. Briefly, the homo- and heterodimers of NF-*κ*B/Rel components assemble inactive complexes with I*κ*B proteins in the cytoplasm. After stimulation by upstream kinases, IKK*α*/*β* kinases phosphorylate I*κ*B proteins which are subsequently ubiquitinated and degraded in proteasomes. Afterwards, dissociated NF-*κ*B/Rel dimers translocate to nuclei where they transactivate the expression of hundreds of genes. This is the basic principle of NF-*κ*B function but the NF-*κ*B pathway can be controlled at several levels which means that NF-*κ*B signaling is a highly context-dependent process.

Recently, Tieri et al. [26] applied a network analysis to reveal the interactome map of NF-*κ*B signaling pathway. They observed that the integrated network affecting NF-*κ*B activation consisted of 622 proteins whereas the NF-*κ*B signaling was able to regulate the expression of 426 proteins of which 49 proteins established a feedback loop controlling NF-*κ*B activity. Many of the upstream signaling pathways converging in the regulation of IKK complex and directly NF-*κ*B components have been characterized [27–29]. The upstream pathways were earlier classified into canonical and noncanonical pathways but currently, it seems that this scenario is not adequate due to intense crosstalk between different signaling pathways. Moreover, IKK complex has many NF-*κ*B-independent, context-dependent functions, for example, in immunity and cancer [30]. Posttranslational modifications of NF-*κ*B components are a potent way to control their transactivation efficiency [31]. Thus, modifications such as phosphorylation, acetylation, methylation, sulfhydration, sumoylation, and ubiquitination can regulate the duration and capacity of transactivation and possibly also the dimerization of NF-*κ*B/Rel components and their recruitment of proper coregulators to specific DNA sites [31–33]. Context-dependent factors affect the specificity which links different stimuli to the activation of relevant NF-*κ*B-driven target genes. For instance, NF-*κ*B signaling generates different responses in acute and chronic inflammation [34]. It is most likely that context-dependent factors control the responses of NF-*κ*B signaling with respect to apoptosis, autophagy, and activation of inflammation. 

## 2. IKK-NF-*κ*B Axis Is a Context-Dependent Regulator of Autophagy

There is a substantial literature indicating that NF-*κ*B signaling and autophagy are reciprocally involved in the control of cellular survival and any dysfunction in this crosstalk can evoke detrimental effects. For instance, it is known that a decline in autophagy can stimulate NF-*κ*B-dependent inflammatory responses [35] whereas an increase in autophagy can prevent inflammatory responses [36]. On the other hand, the inhibitors of NF-*κ*B signaling are effective activators of autophagocytosis, for example, AMPK [37] and SIRT1 signaling [38, 39]. However, there is mounting evidence that NF-*κ*B signaling can either activate or inhibit autophagocytosis in a context-dependent manner (see below). Moreover, several proteins, for example, Bcl-2 family members are key players both in the regulation of autophagy and apoptosis indicating that there could be a context-dependent crosstalk via the activation of IKK-NF-*κ*B signaling axis. Recently, Trocoli and Djavaheri-Mergny [40] reviewed the complex interplay between NF-*κ*B signaling and autophagy in cancer formation.

### 2.1. IKK Complex Controls Autophagy and Vice Versa via Feedback Response

**Figure 1:**
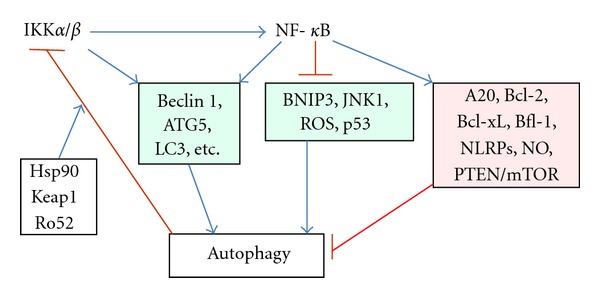
The context-dependent regulation of autophagy via the functional interactions between IKK-NF-*κ*B signaling axis and autophagy. IKK*α*/*β* and NF-*κ*B can increase the expression of Beclin 1 and other autophagy-related proteins and stimulate autophagocytosis. On the other hand, increased autophagy can degrade IKK components and repress autophagy. Hsp90, Keap1, and Ro52 enhance the autophagic degradation of IKK components. In addition, NF-*κ*B signaling increases the expression of autophagy repressors, for example, A20, Bcl-2/Xl, and NLRP receptors. Moreover, NF-*κ*B can suppress many inducers of autophagy, for example, BNIP3, JNK1 and ROS.

Criollo et al. [41] were the first investigators who demonstrated that several autophagic inducers stimulated autophagy via the activation of the IKK complex but without any involvement of NF-*κ*B signaling in several cell lines. They also observed that starvation and rapamycin did not induce autophagy in the livers of mice carrying a conditional knockout of IKK*β*. They did not reveal any exact mechanism but they noted that the IKK complex promoted autophagy by (i) activating AMPK, (ii) inhibiting mTOR, and (iii) depleting nuclear p53, all of which are crucial inducers of autophagy. On the other hand, Comb et al. [42] demonstrated that cellular starvation induced the expression of *Atg5*, *BECN1, *and *LC3*, important autophagy genes, in the IKK*α* and IKK*β*-dependent manner but independently of NF-*κ*B signaling. Interestingly, starvation stimulated the expression of NF-*κ*B-dependent antiapoptotic genes, for example, *Bnip3* and *cIAP2*. These studies indicate that in some circumstances, I*κ*B kinases can stimulate canonical autophagy and induce the expression of autophagy-related genes without NF-*κ*B transcription factors (Figure 1). Recently, Comb et al. [43] revealed that the regulatory subunit of PI3K, p85*α*, is an IKK phosphorylation target in cellular starvation inducing the transient feedback inhibition of PI3K/Akt signaling and subsequently triggering autophagy via the inhibition of mTOR activity. It is important to emphasize here that the autophagy activated by the IKK complex in acute stress, extending up to some hours, was transient, probably due to the effective feedback inhibition.

Hsp90 chaperone is a major stability factor for the activation of IKK signalosome [44–46]. It is known that acute cellular stress can trigger the dissociation of Hsp90 from the IKK complex which inhibits NF-*κ*B signaling [46, 47]. Interestingly, Qing et al. [48] demonstrated that the treatment of cells with geldanamycin, an inhibitor of Hsp90, induced the degradation of IKK*α* and IKK*β* proteins via autophagy and neither ubiquitination nor proteasomes were involved in the degradation of the IKK complex. Moreover, they also revealed that geldanamycin stimulated the autophagic degradation of NIK, a key kinase activating IKK*α* in the noncanonical NF-*κ*B pathway [49]. These studies indicate that Hsp90 has a crucial role in the control of signaling via the IKK-NF-*κ*B axis, that is, acute insults trigger IKK-mediated autophagy and block NF-*κ*B-driven transcription whereas prolonging the stress will stimulate the autophagic degradation of the IKK complex, thus, preventing apoptotic cell death. Niida et al. [50] observed that the excessive activation of IKK*β* could trigger its monoubiquitination by Ro52, an E3 ubiquitin ligase, which subsequently enhanced the autophagic degradation of IKK*β* (Figure 1). Moreover, Keap1, another E3 ligase, can interact with IKK*β*, but not with IKK*α*, and thus induce the autophagic degradation of IKK*β* which consequently inhibits NF-*κ*B signaling [51]. In conclusion, it seems that the IKK complex and autophagy have a mutual function in acute cellular stress, that is, at its onset, IKK-induced autophagy ensures the energy supply and supports housekeeping but later, autophagic degradation of the IKK complex prevents the excessive activation of IKKs and their harmful responses, such as tumorigenesis and inflammation (Figure 1). 

### 2.2. NF-*κ*B Activation Stimulates Autophagy

 There is an interesting context-dependent activation of autophagy between the IKK complex and its downstream target, the NF-*κ*B system. Occasionally, the IKK complex can trigger autophagy without involvement of NF-*κ*B system (Section 2.1.) whereas under other circumstances, NF-*κ*B is a crucial inducer of autophagy. For instance, Nivon et al. [52] demonstrated that NF-*κ*B signaling triggered autophagy during the heat shock recovery period and thus supported cellular survival. Inhibition of NF-*κ*B activation blocked the autophagic response and augmented cell death after a heat shock stress. Recently, they revealed that the NF-*κ*B signaling induced by heat stress increased the expression level of BAG3-HspB8 complexes which enhanced the autophagic uptake and elevated the clearance of irreversibly damaged proteins [53]. It is known that the BAG3-HspB8 chaperone complex targets misfolded proteins for macroautophagy [54]. This implies that NF-*κ*B signaling has an important role in the maintenance of protein quality during heat shock. Excessive heat shock can also induce an atypical activation of NF-*κ*B signaling through a thermolabile dissociation of the NF-*κ*B complex without any phosphorylation of I*κ*B proteins and thus no involvement of the IKK signalosome is necessary [55].

 Copetti et al. [56] demonstrated that the p65/RelA component can induce the transcription of the *BECN1* gene (Beclin 1) and thus stimulate autophagy in T-cells. They identified some conserved NF-*κ*B binding sites both in the promoter and the first intron of the *BECN1* gene in mice and humans. Copetti et al. [56] observed that p65 regulated Beclin 1 expression in several cell types and consistently enhanced the induction of autophagy. Their study indicated that the *BECN1 *gene is one of the target genes of NF-*κ*B signaling. Bhatnagar et al. [57] revealed that the proinflammatory cytokine TWEAK, a well-known NF-*κ*B-dependent inducer of atrophy in skeletal muscles [58], could stimulate the expression of several autophagy genes, for example, *BECN1*, *LC3B*, *Atg5*, in C2C12 myotubes and it also activated autophagocytosis. They also confirmed that the TWEAK-provoked response was mediated by NF-*κ*B signaling. It is known that TWEAK can stimulate NF-*κ*B signaling via TRAF6 and induce a prolonged activation of NF-*κ*B [59]. TRAF6, an E3 ubiquitin ligase, can also ubiquitinate Beclin 1 in BH3 domain and trigger autophagy [60]. It seems that NF-*κ*B signaling can directly stimulate the expression of autophagy-related genes in order to support cell survival but also organismal life since skeletal muscle atrophy supplies amino acids, for example, in starvation (Figure 1).

Jiang et al. [61] revealed that the downregulation of Hsp90 expression by selenite inhibited the signaling of the IKK/NF-*κ*B pathway and significantly reduced the expression of Beclin 1 which subsequently increased the transition from autophagy to apoptosis. Recently, Romano et al. [62] observed that ionizing radiation stimulated NF-*κ*B signaling via the activation of FKBP51 in malignant melanoma cells. Subsequently, the activation of NF-*κ*B protected from apoptotic cell death by inducing XIAP expression and promoting BAX degradation by autophagy. The inhibition of FKBP51 clearly promoted apoptosis in irradiated melanoma cells. These studies indicate that NF-*κ*B-dependent autophagy can prevent apoptotic cell death. However, there are several reports which have demonstrated that the NF-*κ*B-dependent activation of autophagy could lead to autophagic cell death [63–65]. Currently, it is a matter of debate whether autophagy can be the mechanism of cell death [66].

### 2.3. NF-*κ*B Signaling Indirectly Suppresses Autophagy

 There are many observations which indicate that under certain circumstances the NF-*κ*B system can also mediate the suppression of autophagy. For instance, Djavaheri-Mergny et al. [67] observed that NF-*κ*B signaling inhibited the autophagy induced by TNF-*α* in different cell types. They revealed that NF-*κ*B signaling activated mTOR kinase which is the major inhibitor of autophagy. Moreover, suppression of NF-*κ*B signaling in this model stimulated the expression of Beclin 1 and subsequently triggered autophagy in TNF-*α*-treated cells. This response was mediated by ROS which can context-dependently promote autophagocytosis [68]. Upon TNF-*α* treatment, the activation of IKK*α*/*β* can repress TSC complex [69, 70], a potent inhibitor of mTOR, and thus induce the activation of autophagy. On the other hand, NF-*κ*B signaling, induced by TNF-*α*, can inhibit the expression of PTEN, a dual-specificity phosphatase that inhibits insulin/Akt pathway [71, 72]. The insulin/Akt pathway is a potent activator of mTOR and thus NF-*κ*B signaling can inhibit autophagy by stimulating this homeostatic pathway (Figure 1). Interestingly, signaling via insulin/IGF pathway can accelerate the aging process [73]. Schlottmann et al. [74] demonstrated that the prolonged activation of NF-*κ*B signaling inhibited the expression of *BECN1* and *Atg5* genes and repressed autophagy in macrophages. Interestingly, it seems that the activation of NF-*κ*B signaling in acute stress can provoke autophagy (Section 2.2) whereas a delayed activation can suppress autophagy. Probably, this is a feedback regulation which could prevent cells from autophagic cell death. It is known that NF-*κ*B signaling can control the expression of several, well-known activators and inhibitors of autophagy and in that way indirectly and context-dependently regulate autophagy (Figure 1).

#### 2.3.1. NF-*κ*B Signaling Enhances the Expression of Autophagy Inhibitors

**Figure 2:**
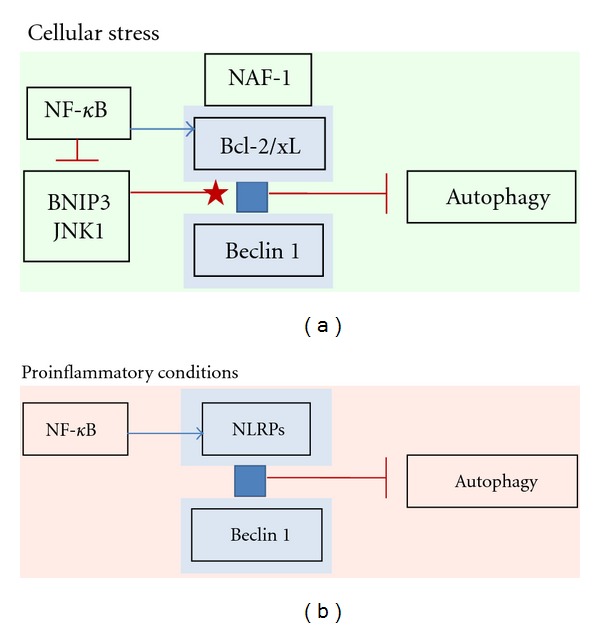
NF-*κ*B signaling controls autophagy via the Beclin 1 interactome. (a) During cellular stress, NF-*κ*B increases the expression of Bcl-2/xL proteins which bind via the NAF-1 to the endoplasmic reticulum and repress the function of the Beclin 1 initiation complex. On the other hand, in some circumstances, NF-*κ*B can inhibit the expression of BNIP3 and oppose the activity of JNK1 which normally dissociates the Beclin 1/Bcl/xL complex (star-head). (b) In proinflammatory conditions, NF-*κ*B signaling increases the expression of several NLRP receptors which can bind to Beclin 1 and this can inhibit autophagocytosis.

 Beclin 1 network is a crucial regulator of autophagocytosis but also apoptosis [13, 18, 75]. Beclin 1 protein contains three conserved domains, that is, BH3 (Bcl-2-homology-3), ECD (evolutionarily conserved domain) and CCD (coiled-coil domain) through which it binds a number of proteins to create a functional network called the Beclin 1 interactome. For instance, Beclin 1 binds with Bcl-2 and Bcl-xL, which are the major antiapoptotic proteins and thus the Beclin 1 interactome can control both autophagy and apoptosis (Figure 2). Binding of Bcl-2 and Bcl-xL to Beclin 1 protein inhibits the formation of the initiation complex and subsequently blocks autophagocytosis. However, the phosphorylation of Bcl-2 by JNK1 [16] and Beclin 1 by DAPK [76] can dissociate the interaction between Beclin 1 and Bcl-2/xL which supports autophagocytosis. The versatile balance between the proteins binding to Bcl-2/xL and Beclin 1 controls the activity of autophagy [18]. For instance, the overexpression of Bcl-xL delays the onset of autophagy [77] whereas downregulation of Bcl-2 stimulates autophagy [78]. Interestingly, it is known well that NF-*κ*B signaling is a potent inducer of Bcl-2 and Bcl-xL transcription [79–83] and thus the expression of Bcl-2/xL inhibits apoptosis but simultaneously it also represses autophagy. Moreover, NF-*κ*B signaling stimulates the transcription of Bfl-1/A1, a Bcl-2 family member and a Beclin 1 binding partner, which also negatively regulates autophagy [84, 85].

Recently, Chang et al. [86] characterized the Bcl-2-interacting protein NAF-1 (nutrient-deprivation autophagy factor-1) in endoplasmic reticulum, which is required for the interaction between Beclin 1 and Bcl-2. It is known that Bcl-2 inhibits the Beclin 1-mediated autophagy only if it is present in ER. Chang et al. [86] demonstrated that NAF-1 also interacted with IP3 receptor and mediated the Bcl-2-induced regulation of ER calcium homeostasis. BH3 protein BIK displaced NAF-1 from Bcl-2 and triggered autophagy. There are also reports indicating that the overexpression of Bcl-2 could activate NF-*κ*B signaling via the MEKK-1/IKK*β* pathway [87, 88]. This might indicate a positive feedback in the prevention of apoptosis. In conclusion, there is mounting evidence indicating that NF-*κ*B signaling can induce autophagy by inducing the transcription of autophagy genes (Section 2.2) but on the other hand, it can repress autophagy by stimulating the expression of antiapoptotic, Bcl-2 family genes. It seems that NF-*κ*B signaling controls the crosstalk between autophagy and apoptosis in a context-dependent manner. 

The ubiquitination of Beclin 1 is a potent enhancer of autophagy [60]. Shi and Kehrl [60] demonstrated that LPS as well as many other stimuli of autophagy in murine macrophages, for example, IL-1, IFN*γ*, and amino acid starvation, induced Lys-117 ubiquitination of Beclin 1. They observed that TRAF6 was able to bind to Beclin 1 and it triggered a K63-linked ubiquitination at the BH3 site of Beclin 1 protein. They also revealed that ubiquitination facilitated the oligomerization of Beclin 1 and thus enhanced autophagy, probably preventing the formation of inhibitory complexes between Bcl-2/xL and Beclin 1. Shi and Kehrl [60] also observed that A20, a ubiquitin-editing enzyme [89], provoked the deubiquitination of Beclin 1 and in that way inhibited autophagic capacity of Beclin 1. Interestingly, A20 deubiquitinase is a target gene of NF-*κ*B transactivation [90] but on the other hand, the expression of A20 establishes a negative feedback mechanism to shut down the activation of NF-*κ*B system [91, 92]. The activation of the NF-*κ*B signaling pathway, for example, by TNF and IL-1 receptors, involves several regulatory steps mediated by K63 ubiquitination at the level of upstream kinases and inhibitory I*κ*B proteins [89, 91]. For instance, A20 inhibits the activity of IKK complex [93]. A20 is a well-known inhibitor of NF-*κ*B-mediated inflammatory responses occurring in Crohn's disease, rheumatoid arthritis, and psoriasis [89]. It seems that A20 and its coactivator ABIN-1 [94] could be potent inhibitors of Beclin 1 function enhancing its deubiquitination and thus supporting its complex formation with Bcl-2/xL proteins during the chronic inflammation.

Recently, Jounai et al. [95] observed that Beclin 1 can interact with the members of NLR family of inflammasome receptors, for example, NLRC4, NLRP3, NLRP4, and NLRP10. They revealed that the evolutionarily conserved domain (ECD) of Beclin 1 could physically bind to the NACHT domain of NLR proteins. In particular, the NACHT of NLRP4 induced a tight binding with Beclin 1. Jounai et al. [95] also revealed that NLRP4 and NLRC4 inhibited the autophagosome formation whereas NLRP4 could also repress the maturation of autophagosomes to autolysosomes. NLR proteins are important intracellular danger sensors assembling inflammasomes which in turn activate inflammatory caspases to cleave the proforms of IL-1*β* and IL-18 into the mature, secreted cytokines [4, 96]. Bauernfeind et al. [97] demonstrated that the activation of inflammasomes was dependent on the priming phase in which NF-*κ*B signaling induced the expression of NLRP3 and the proforms of IL-1*β* and IL-18 in order to assemble inflammasomes. Currently, it is not known whether other NLR receptors require the priming stage to facilitate inflammasome activation. However, it seems that inflammasome receptors can bind to Beclin 1 and thus repress autophagy. On the other hand, Shi et al. [98] demonstrated that the activation of autophagy by inflammatory signals induced the ubiquitination of ASC adapter of inflammasomes and subsequently triggered their degradation via p62-mediated selective autophagy. This indicates that autophagy can repress the overwhelming inflammation and imply that a decline in autophagic capacity can generate chronic inflammatory conditions both during aging process and age-related diseases (Section 3). There are also reports that antiapoptotic proteins Bcl-2/xL could bind to NLRP1 protein and thus inhibit its inflammasome function [99, 100]. These observations imply that antiapoptotic proteins Bcl-2/xL can also regulate inflammation, in addition to autophagocytosis. 

There is a substantial literature indicating that ROS and oxidative stress can control autophagocytosis [68, 101, 102]. Moreover, inducible nitric oxide synthase (iNOS), induced by NF-*κ*B activation, stimulates the production of nitric oxide (NO) which has a crucial role as a messenger molecule but its dysfunction generates nitrosative stress and provokes many diseases [103]. Recently, Sarkar et al. [104] demonstrated that NO could inhibit autophagy by S-nitrosylating and thus inhibiting the activities of IKK*β* and JNK1, potent inducers of autophagy. The inhibition of JNK1 by NO reduces the phosphorylation of Bcl-2 and in that way increases the formation of inhibitory Bcl-2/Beclin 1 complexes. The S-nitrosylation of IKK*β* reduces its capacity to activate AMPK*α*, an important inducer of autophagy by activating ULK1 and repressing mTOR [105]. These studies indicate that cellular stress can repress autophagy via the reactive nitrogen species induced by NF-*κ*B signaling.

#### 2.3.2. NF-*κ*B Signaling Represses the Expression of Autophagy Activators

 The interactome of Beclin 1 contains several binding factors, for example, Bcl-2 and Bcl-xL (Section 2.3.1), but the Beclin 1 complexes can be disrupted by BH3 domain containing proteins, for example, BNIP3 and NIX/BNIP3L, which bind to Bcl-2 and Bcl-xL proteins and subsequently activate the formation of the Beclin 1/hVps34/hVps15 interaction, an initiation complex in the process of autophagocytosis [17]. Hypoxia is a potent inducer of BNIP3 and NIX expression which in particular, enhances the autophagic uptake of mitochondria, a process called mitophagy [106]. It is known that BNIP3 and NIX represent a survival mechanism in moderate stress but excessive insults contribute to mitochondrial damage and consequently to cell death through the induction of apoptosis, for example, in the hypoxic myocardium [106, 107]. Several transcription factors regulate the transcription of *Bnip3* gene, for example, HIF-1*α*, NF-*κ*B, E2F1, FoxO3, p53, and Sp1 [107–109]. Moreover, the promoter of *Bnip3* contains several GC-rich regions and is hypermethylated and silenced in many cancers. Kirshenbaum and coworkers demonstrated that the binding of p65 component of the NF-*κ*B complex induced the transcriptional repression of BNIP3 expression in rat ventricular myocytes [109–111] (Figure 2). Under physiological conditions, the NF-*κ*B complex interacts with HDAC1, histone deacetylase 1, and prevents the binding of E2F1 to the adjacent sites in *Bnip3* promoter. In hypoxic conditions or during the deficiency of NF-*κ*B, binding of E2F1, HIF-1*α*, and FoxO3 factors stimulate autophagy which can lead to cell death. Moreover, p53 can also suppress the expression of BNIP3 by binding to the *Bnip3* promoter and recruiting the corepressor Sin3a [108]. These studies indicated that the expression level of BNIP3 is a crucial regulator of autophagy through Beclin 1 interactome. They also revealed that autophagy is under the epigenetic regulation via BNIP3 expression.

Starvation is the most crucial, evolutionarily conserved inducer of autophagy [112]. Wei et al. [16] demonstrated that starvation triggered the phosphorylation of Bcl-2 at several residues and induced its dissociation from Beclin 1 which provoked autophagocytosis. They observed that phosphorylation was caused by the stress-activated JNK1. Many other stress-related factors can also activate autophagy through the JNK1 pathway, stimulating the dissociation of Bcl-2/Beclin 1 complex, for example, ceramides [113]. There is substantial evidence that NF-*κ*B signaling suppresses the JNK1 signaling cascade and thus can protect cells from JNK1-induced apoptosis [114–117]. Several studies have indicated that NF-*κ*B signaling can induce the expression of antioxidants and antiapoptotic proteins which prevent the prolonged activation of JNK1 signaling for example, during the TNF-*α* exposure. For instance, NF-*κ*B signaling stimulates the expression of Gadd45*β* which inhibits the activity of MKK7, an upstream kinase of JNK1 [114]. Moreover, A20 and XIAP, which are also induced by NF-*κ*B, inhibit the TNF-*α*-mediated JNK1 activation [115, 116]. 

It is well known that ROS and redox signaling can increase autophagocytosis via many different mechanisms, in addition to JNK1 regulation [68, 101, 102]. For instance, ROS can oxidize cysteine-rich domains of ATG4 and Rubicon and thus enhance autophagy [68, 102]. Currently, the exact mechanisms still await to be characterized. Recently, Lipinski et al. [118] demonstrated that ROS can activate type III PI3 kinase, also known as hVSP34 which is a Beclin 1-interacting protein and the major enhancer of autophagy [12]. Regulation of antioxidant gene expression is one of the major functions of NF-*κ*B signaling [119] and thus NF-*κ*B activity can repress autophagy by reducing the presence of ROS although in many contexts it can protect cells from injuries induced by excessive ROS production. 

There are several aspects indicating that autophagy is a tumor suppressor mechanism, for example, Beclin 1 is a haploinsufficient tumor suppressor protein, and chronic inhibition of autophagy can increase carcinogenesis [120, 121]. Moreover, p53 can have both activating and inhibiting effects on autophagy, that is, nuclear p53 stimulates autophagy but in cytoplasm, p53 represses autophagy [120, 122]. On the other hand, there is mounting evidence pointing out that NF-*κ*B signaling can induce tumorigenesis, either directly or via inflammation [123]. Whether or not this is linked to the capacity of NF-*κ*B to repress autophagy still remains to be revealed. Interestingly, several studies have indicated that p53 and NF-*κ*B have many antagonistic functions [124]. Nuclear p53 is a potent inducer of autophagy since it can transactivate many activators of autophagy, for example, DRAM1, DAPK-1 and SESN2 [125]. However, the exact mechanism by how NF-*κ*B-dependent signaling could inhibit the transcriptional activity of p53 is not known. 

In conclusion, there is substantial literature which shows that NF-*κ*B signaling can either stimulate or inhibit autophagocytosis. It is clear that the control of autophagy by NF-*κ*B system is a context-dependent process which is closely linked together apoptosis and autophagy. It seems that NF-*κ*B signaling, which is a potent antiapoptotic factor and inducer of inflammatory defence, could also have harmful consequences by inhibiting autophagocytosis. 

## 3. Decline in Autophagy with Aging: Potential Role of NF-*κ*B Signaling

### 3.1. Hallmarks of Inflammaging and Cellular Senescence

 During the last decade, mounting evidence has revealed that there is a clear imbalance between adaptive and innate immunity in mammals during the aging process [126, 127]. Immunosenescence of adaptive immunity system enhances the activation of innate immunity responses which generates a proinflammatory phenotype, called inflammaging [126]. The major characteristics of inflammaging are the increased expression of genes associated with inflammation in tissues [128, 129] and augmented levels of cytokines, for example, IL-6 and TNF-*α*, in serum [130, 131]. These observations agree with earlier studies indicating that the NF-*κ*B system is activated with aging in many tissues [132–135]. Interestingly, inflammaging is also linked to a clear decline in autophagy [6–8, 136] as well as apoptosis [137–141]. Moreover, it seems that the aging process *in vivo* also involves the appearance of senescent cells which are resistant to apoptotic cell death [142–145]. Campisi et al. [146] have revealed that cellular senescence is linked to a proinflammatory phenotype called the senescence-associated secretory phenotype (SASP). Recent studies have indicated that the formation of this condition is stimulated by the activation of NF-*κ*B signaling [147]. Kang et al. [148] demonstrated that impairment of autophagy induces a premature senescence in normal human fibroblasts and on the other hand, replicatively senescent cells expressed reduced levels of autophagy proteins. However, autophagy can facilitate oncogene-induced senescence [149] and trigger autophagic cell death by increasing the expression of Noxa which is able to displace Bcl-2 family members from the Beclin 1 complex [150]. Impaired autophagy supports the accumulation of waste materials into cells, called the “garbage-can hypothesis” by Brunk and Terman [151]. Recent studies have indicated that the decline in autophagy and impaired housekeeping can stimulate NF-*κ*B signaling and generate chronic inflammation via the activation of inflammasomes [4, 5, 152]. In particular, oxidative stress, which increases with aging, is a potent inducer of inflammasomes [153]. 

### 3.2. Age-Related Decline in the Expression of Autophagy Proteins 

The mechanism of age-related decline in autophagy is still unclear. Genome-wide analysis of Lipinski et al. [118] indicated that autophagy could be transcriptionally downregulated during normal aging process in the human brain. Their study also revealed that aging could decrease the activity of type III PI3K (hVPS35), which is a crucial factor of Beclin 1 interactome during the nucleation phase of autophagy. Protein-protein interaction analysis revealed that RIP1 and PKC*ζ* kinases and p62/sequestosome could be involved in the regulation of autophagy with aging. All of these three proteins are associated with NF-*κ*B signaling and autophagy [154–156]. Hua et al. [157] observed a significant decrease in the protein levels of Beclin 1 and ATG5 whereas that of insoluble p62 was elevated, probably indicating its binding to protein aggregates in the hearts of old mice. Moreover, aging decreased the activity of AMPK whereas mTOR activity was increased, both of these changes could reduce autophagic activity. Wohlgemuth et al. [158] reported that the protein levels of ATG7, ATG9, and LAMP1 decreased but that of Beclin 1 was unaffected in old rat hearts. In general, these studies indicate that the expression of several autophagy proteins seems to be decreased during organismal aging as well as under cellular senescence *in vitro* conditions [148]. However, the changes are small and tissue-specific and not repeatable in different studies which implies that many other factors in addition to the expression level can contribute to the decline in the autophagic process in conjunction with aging.

### 3.3. NF-*κ*B Signaling Inhibits Autophagy via Beclin 1 Interactome during Aging Process

The regulation of the Beclin 1 interactome is a crucial control mechanism in the initiation of autophagy [13, 18]. As described earlier (Section 2.3.1), transcription factor NF-*κ*B is an important transactivator of Beclin 1 itself as well as of several other binding partners, for example, Bcl-2, Bcl-xL, and NLRP4. Normally, Beclin 1 protein is complexed with Bcl-2/xL and autophagy is inhibited but the balance is dynamic and can be controlled by the availability of the different binding partners. Many reports have demonstrated that aging is linked to an increase in the expression of antiapoptotic Bcl-2 and Bcl-xL proteins, in particular in brain [159, 160]. Moreover, Satou et al. [161] observed that the immunoreactivity of Bcl-2 protein within neurons increased with the severity of Alzheimer's disease. It also seems that oxidative stress and accumulation of lipofuscin could enhance the expression of Bcl-2 in neurons with aging [159, 160]. Xu et al. [162] demonstrated that the intracerebroventricular injection of proinflammatory cytokines, TNF-*α* and IFN*γ*, markedly increased the expression of Bcl-2 in neurons of cortex and hippocampus. Interestingly, the response was age-related being clearly more prominent in the brains of old mice. This implies that inflammation, probably via the stimulation of NF-*κ*B signaling, is a significant contributor to the decline of apoptosis and autophagy during aging.

 Several studies have revealed that the expression of Bcl-2/xL proteins is clearly increased in senescent cells *in vitro*, simultaneously with the augmentation of apoptosis resistance [145, 163]. Wang [163] observed that serum deprivation reduced the level of Bcl-2 in young, proliferating human fibroblasts whereas in senescent cells, the expression level of Bcl-2 remained unchanged. This agrees with the observations that the capacity for autophagy is impaired in human senescent fibroblasts [148]. Moreover, the overexpression of Bcl-2 reduces the lifespan of cultured human fibroblasts [164] as well as induces cellular senescence in human carcinoma cells [165]. Lee et al. [166] demonstrated that the inhibition of JNK1 activity and subsequently the reduction in the level of Bcl-2 phosphorylation, the signal for autophagy (Section 2.3.1), induced cellular senescence both in human fibroblasts and carcinoma cells. This indicates that the autophagy via JNK/Bcl-2 phosphorylation is essential in the prevention of cellular senescence. These studies imply that Bcl-2/xL proteins have a crucial role with aging in the decline of apoptotic capacity as well as autophagy via the stabilization of Beclin 1 complexes. In view of the important function of NF-*κ*B signaling in the generation of cellular senescence [147], it seems plausible that the overexpression of Bcl-2/xL has a major role in the appearance of the senescent phenotype.

A decline in autophagy with aging can also increase ROS production and stimulate the expression of inflammasome components via NF-*κ*B signaling (Section 2.3.1). Interestingly, many of the inflammasome receptors can interact with Beclin 1, for example, NLRC4, NLRP3 and NLRP4, and inhibit autophagocytosis [95]. The Beclin 1 interactome also contains HMGB1 proteins, enigmatic proteins which can regulate (i) genome function in nuclei, (ii) NF-*κ*B transactivation, (iii) autophagy in cytoplasm, and (iv) inflammatory responses in extracellular space [167–170]. Tang et al. [171] demonstrated that HMGB1 could directly bind to Beclin 1 which displaced the inhibitory Bcl-2 protein from the complex and subsequently activated autophagy. HMGB1 is evolutionarily conserved and contains sequence homology with Beclin 1 which implies that it controls autophagocytosis. Inflammation and severe stress translocate the nuclear HMGB1 to the cytoplasm and extracellular space. ROS and oxidative stress oxidize the cysteine residues of HMGB1 which is required for the cytoplasmic translocation of HMGB1 and its binding to Beclin 1 and the subsequent stimulation of autophagy [168]. On the other hand, the activation of NLRP3 inflammasomes triggers the secretion of HMGB1 protein into the extracellular space where it can bind several immune receptors and thus stimulate inflammatory responses [172]. HMGB1 is an architectural factor in genome, for example, assisting in the binding of NF-*κ*B complexes to DNA [167]. There are age-related, tissue-specific changes in the expression of nuclear HMGB1 (previously called HMG-1), for example, the level of HMGB1 clearly decreases in brain (neurons), heart, lung, and thymus [173, 174]. In contrast, the overexpression of HMGB1 is one of the hallmarks of cancer [175]. In this scenario, it seems that autophagy and inflammation are evolutionarily linked to each other to defend the host organism.

### 3.4. NF-*κ*B Signaling Controls Autophagy via ROS Balance

In 1956, Harman [176] presented the free radical theory of aging which states that oxygenderived free radicals attack cellular constituents causing structural damage and impairing housekeeping functions. This theory has received substantial support since it was appreciated that the level of ROS-induced damage augments with aging. However, ROS are signaling molecules, for example, stimulating autophagy (Section 2.3.2) and it is known that the enhancement of autophagy increases the lifespan [136, 177]. Hekimi et al. [178] have proposed a gradual ROS response hypothesis, which maintains that low ROS levels activate signaling pathways which protect cells against different insults. The activation of autophagocytosis could represent that kind of protective response. However, with increases in the duration and input of stress, the accumulation of ROS could become detrimental and induce toxic responses. This theory could explain the apparently contradictory evidence that is, that a low level of oxidative stress can extend the lifespan in animal experiments [179]. Recently, Morgan and Liu [119] reviewed the extensive crosstalk between ROS and NF-*κ*B signaling. For instance, NF-*κ*B signaling induces the expression of major antioxidants, for example, superoxide dismutases, thioredoxins, heme oxygenase, and glutathione peroxidase-1. On the other hand, NF-*κ*B stimulates the expression of many ROS-producing enzymes, for example, NADPH oxidase-2, inducible NOS, and xanthine oxidase. ROS can also directly control the activity of NF-*κ*B signaling in a cell type-specific manner [119]. Moreover, Bauernfeind et al. [180] demonstrated that the NF-*κ*B-provoked induction of NLRP3 expression, an inhibitory binding partner of Beclin 1 (Section 2.3.1), was highly dependent on the presence of ROS. In this way, ROS could inhibit the function of Beclin 1 initiation complex and thus repress autophagy. It seems that NF-*κ*B signaling has a crucial role in the context-dependent regulation of autophagy. Currently, it is not known whether a low-grade inflammatory state with increased ROS levels could generate some feedback responses in the NF-*κ*B signaling which might suppress autophagocytosis with aging. 

### 3.5. Decline in Autophagy Activates IKK*β*/NF-*κ*B Signaling in Hypothalamus and Provokes Metabolic Syndrome

Metabolic syndrome is a major age-related disorder involving obesity, hypertension, diabetes, and cardiovascular diseases. Zhang et al. [181] demonstrated that overnutrition, a common cause of metabolic syndrome, activated IKK*β*/NF-*κ*B signaling in hypothalamic neurons and caused central insulin and leptin resistance. Further studies have revealed that excessive nutrition and systemic inflammation induced oxidative and endoplasmic stresses and activated NF-*κ*B signaling in hypothalamus which subsequently triggered metabolic inflammation and provoked obesity and hypertension [182, 183]. Recently, Meng and Cai [35] demonstrated that the tissue-specific inhibition of autophagy in hypothalamus stimulated IKK*β*/NF-*κ*B signaling and induced inflammatory changes which were associated with the development of obesity and systemic insulin resistance. They also observed that the ablation of IKK*β* abrogated the effects of deficient autophagy. These studies indicate that the decline in autophagy in hypothalamic neurons can lead to metabolic dysfunctions in many tissues and ultimately induce metabolic syndrome. Transgenic and knockout models have revealed complex, tissue-specific functions of NF-*κ*B signaling [184]. Moreover, aging responses are tissue-specific [185] and thus it needs to be clarified whether aging controls the tissue-specific crosstalk between autophagy and NF-*κ*B system. 

## 4. Conclusions

The NF-*κ*B signaling system and autophagic degradation pathway are evolutionarily conserved, major cellular survival mechanisms. It is most likely that they have engaged in a close crosstalk with each other although its detailed characterization has remained elusive. There is emerging evidence indicating that the reciprocal regulation is highly dependent on the cellular context which emphasizes their fundamental role in host defence. Moreover, it seems that autophagy and apoptosis are controlled in cooperation with certain common regulatory proteins, for example, Bcl-2, Bcl-xL and BNIP3. It is also known that autophagy is an important regulator of inflammatory responses, in particular via the inflammasomes. Recent studies have revealed that the Beclin 1 interactome is a key player which controls autophagic initiation but it has also crucial effects on the function of apoptosis and inflammasomes. Interestingly, the NF-*κ*B transcription factor directly regulates the transactivation of Beclin 1 and many other components of the Beclin 1 interactome. Moreover, NF-*κ*B signaling controls the level of ROS and oxidative stress which are potent activators of autophagy. One puzzling aspect is that IKK*α*/*β*, close upstream kinases of NF-*κ*B signaling, can in certain contexts stimulate autophagy without any activation of NF-*κ*B signaling. 

A decline in autophagic capacity is a hallmark of the aging process. Aging is also associated with increased apoptosis resistance and augmented inflammatory responses. Currently, it is not known whether the regulation mechanisms of autophagy, apoptosis, and inflammasomes are linked to each other but clearly they act in concert to aggravate the aging phenotype and expose tissues to the danger of age-related degenerative diseases. There are some indications that the Beclin 1 interactome and increased oxidative stress may be critically involved in the aging process. Several studies have revealed that NF-*κ*B signaling is enhanced with aging which could explain the appearance of a low-grade inflammatory phenotype as well as the decline in autophagy and increased apoptosis resistance. Intriguingly, it is likely that the highly chronic nature of the aging process affects the regulation of autophagy by NF-*κ*B signaling in a context dependent manner that is, impairing autophagy and inducing a proinflammatory aging phenotype. 

## Abbreviations

A20: Ubiquitin-editing enzyme A20
ABIN-1: A20-binding inhibitor of NF-*κ*B1
ASC: Apoptosis-associated speck-like protein containing a CARD
Akt: Protein kinase B
AMPK: AMP-activated protein kinase
ATG: Autophagy-related protein
BAD: Bcl-2-associated death promoter protein
BAG3: Bcl2-associated athanogene 3
BAX: Bcl2-associated X protein
Bcl-2: B-cell lymphoma-2
Bcl-3: B-cell lymphoma-3
Bcl-xL: B-cell lymphoma-extra large
BECN1: Beclin 1 gene
Bfl-1: Bcl2-related protein A1
BH3: Bcl2-homology-3 domain
BIK: Bcl2-interacting killer
BNIP3: Bcl-2 adenovirus E1B 19 kDa-interacting protein 3
CCD: Coiled-coil domain
c-IAP2: Apoptosis inhibitor 2
DAPK: Death-associated protein kinase 1
DRAM1: Damage-regulated autophagy modulator 1
E2F1: E2F transcription factor 1
ECD: Evolutionarily conserved domain
ER: Endoplasmic reticulum
FKBP51: FK506-binding protein 5
FoxO3: Forkhead box O 3
Gadd45*β*: Growth arrest- and DNA damage-inducible gene 45*β*
HDAC1: Histone deacetylase 1
HIF-1*α*: Hypoxia-inducible factor-1*α*
HMGB1: High mobility group box 1
Hsp90: Heat shock protein 90
HspB8: Heat-shock 22-kD protein 8
hVPS34: Vacuolar protein sorting 34 (mammalian homologue)
I*κ*B: Inhibitor of *κ*B protein
IFN*γ*: Interferon *γ*
IGF: Insulin-like growth factor
IKK*α*/*β*: Inhibitor of nuclear factor-*κ*B kinase *α*/*β*
IL: Interleukin
iNOS: Inducible nitric oxide synthase
IP3: Inositol 1,4,5-triphosphate
JNK1: c-Jun NH(2)-terminal protein kinase 1
Keap1: Kelch-like ECH-associated protein 1
LAMP1: Lysosome-associated membrane protein 1
LC3: Microtubule-associated protein 1A/1B-light chain 3
LPS: Lipopolysaccharide
MEKK-1: Mitogen-activated kinase kinase kinase 1
MKK7: Mitogen-activated protein kinase kinase 7
mTOR: Mammalian target of rapamycin
NACHT: Nucleotide-binding protein domain
NADPH: Nicotinamide adenine dinucleotide phosphate
NAF-1: Nutrient-deprivation autophagy factor-1
NEMO: NF-*κ*B essential modulator
NF-*κ*B: Nuclear factor-*κ*B
NIK: NF-*κ*B inducing kinase
NIX: Bcl-2 adenovirus E1B 19 kDa-interacting protein 3-like
NLR: Nucleotide-binding domain and leucine-rich repeat-containing family of proteins
NO: Nitric oxide
PI3K: Phosphatidylinositol 3-kinase
PKC*ζ*: Protein kinase C*ζ*
PTEN: Phosphatase and tensin homolog
Rel: Proto-oncogene v-Rel homolog
RIP1: Receptor-interacting protein 1
ROS: Reactive oxygen species
SASP: Senescence-associated secretory phenotype
SESN2: Sestrin 2
Sin3a: Sin3, yeast homolog A
SIRT1: Silent information regulator 1
Sp1: Transcription factor Sp1
TNF-*α*: Tumor necrosis factor-*α*
TRAF6: TNF receptor-associated factor 6
TSC: Tuberous sclerosis
TWEAK: TNF-related weak inducer of apoptosis
ULK1/2: Unc-51-like kinase 1/2
XIAP: Inhibitor of apoptosis, X-linked.
